# Isolation of the Sarcoplasmic Reticulum Ca^2+^-ATPase from Rabbit Fast-Twitch Muscle

**DOI:** 10.3390/mps6050102

**Published:** 2023-10-19

**Authors:** Miguel A. Rivera-Morán, José G. Sampedro

**Affiliations:** Instituto de Física, Universidad Autónoma de San Luis Potosí, Avenida Chapultepec 1570, Privadas del Pedregal, San Luis Potosí 78295, Mexico

**Keywords:** SERCA, Ca^2+^-ATPase, trehalose, enzyme stabilization, fluorescent labeling, ATP binding, circular dichroism, P-type ATPase, enzyme purification, ultracentrifugation

## Abstract

The sarcoendoplasmic reticulum Ca^2+^-ATPase (SERCA) is a membrane protein that is destabilized during purification in the absence of calcium ions. The disaccharide trehalose is a protein stabilizer that accumulates in the yeast cytoplasm when under stress. In the present work, SERCA was purified by including trehalose in the purification protocol. The purified SERCA showed high protein purity (~95%) and ATPase activity. ATP hydrolysis was dependent on the presence of Ca^2+^ and the enzyme kinetics showed a hyperbolic dependence on ATP (*K*_m_ = 12.16 ± 2.25 μM ATP). FITC labeling showed the integrity of the ATP-binding site and the identity of the isolated enzyme as a P-type ATPase. Circular dichroism (CD) spectral changes at a wavelength of 225 nm were observed upon titration with ATP, indicating α-helical rearrangements in the nucleotide-binding domain (N-domain), which correlated with ATP affinity (*K*_m_). The presence of Ca^2+^ did not affect FITC labeling or the ATP-mediated structural changes at the N-domain. The use of trehalose in the SERCA purification protocol stabilized the enzyme. The isolated SERCA appears to be suitable for structural and ligand binding studies, e.g., for testing newly designed or natural inhibitors. The use of trehalose is recommended for the isolation of unstable enzymes.

## 1. Introduction

In muscle cells, relaxation of the molecular contractile machinery is mediated by the activity of the sarcoendoplasmic reticulum Ca^2+^-ATPase (SERCA), in which ATP hydrolysis is coupled to Ca^2+^ pumping into the sarcoendoplasmic reticulum (SR), resulting in a decrease in the cytoplasmic Ca^2+^ concentration [1,2,3]. SERCA is a member of the P-type ATPase family, which are integral membrane enzymes that generate ionic/chemical potentials (i.e., primary active transporters) and are therefore thought to be present in all living organisms [4]. Three major groups of Ca^2+^-ATPases have been identified, located in different cellular structures, namely the SR, the Golgi, and the plasma membrane. Interestingly, despite significant amino acid sequence differences (up to ~70%), Ca^2+^-ATPases share a similar three-dimensional (3D) structure and catalytic mechanism (Post-Albers scheme) [3,5,6,7]. Importantly, conserved amino acids are involved in ATP binding and hydrolysis, phosphotransfer, and ion transport [4]. The best-studied calcium pump is fast-twitch-muscle SERCA, (SERCA1a, EC 7.2.2.10), a ~110 kDa enzyme [1,8,9,10,11,12,13]. For example, numerous X-ray diffraction studies of SERCA crystals have been reported, and a wealth of three-dimensional (3D) structures in different catalytic states are now available for the enzyme [14,15,16]. Nevertheless, functional–structural information can still be obtained with other experimental techniques; e.g., previously unrecognized catalytic states have been detected in single-molecule studies using fluorescent labeling [17,18], while cryoelectron microscopy is becoming the method of choice for studying other P-ATPases [19,20,21,22,23].

The isolation of membrane proteins/enzymes while preserving their native structure is of paramount importance for in vitro functional–structural experiments. In this context, the isolation of a P-ATPase in high purity and catalytically active form can help to test interacting molecules identified by molecular docking and molecular dynamics simulations [15,24,25,26]. P-type ATPases are known to be remarkably labile to harsh media conditions; e.g., Ca^2+^-ATPase is known to be sensitive to high centrifugation speeds (i.e., high pressure), the presence of oxidative compounds, a relatively extreme pH and mild heat [27,28,29,30]. Recently, the yeast plasma membrane H^+^-ATPase was isolated in its active hexameric state and at high purity [31]. The existence of the H^+^-ATPase hexamer as a functional–structural state was later confirmed and its three-dimensional (3D) structure determined using cryo-electron microscopy [23]. The isolation of the H^+^-ATPase hexamer was achieved using the disaccharide trehalose [31], a well-known protein stabilizer [32,33] that prevents protein unfolding and monomer dissociation [34,35].

In this work, SERCA was isolated from fast-twitch muscle using a purification protocol based on that used to isolate yeast plasma membrane H^+^-ATPase [31,36]. The purified SERCA was of high purity and exhibited enzyme kinetics and other biochemical properties similar to those reported in the literature. Fluorescence labeling with FITC was used to test the integrity of the SERCA binding site and the identity of the enzyme as a P-type ATPase [37]. The secondary structure of SERCA and the effect of ATP binding were analyzed using circular dichroism (CD) spectroscopy. The results showed that the isolated SERCA is suitable for functional and structural experiments, e.g., to test inhibitors (naturally occurring or designed and synthesized in laboratories) identified by molecular docking and molecular dynamics simulation [15,24,25,26].

## 2. Materials and Methods

### 2.1. Chemicals and Reagents

Sucrose, Trizma base, Folin–Ciocalteu phenol reagent, ethyleneglycol-bis(2-aminoethylether)-*N*,*N*,*N*′,*N*′-tetraacetic acid (EGTA), ethylenediaminetetraacetic acid disodium salt dihydrate (EDTA), deoxycholic acid, trehalose, adenosine triphosphate (ATP), fluorescein isothiocyanate (FITC) and rabbit muscle pyruvate kinase were purchased from Sigma-Aldrich Corp. (St. Louis, MO, USA). Rabbit muscle lactate dehydrogenase was purchased from Roche (Basel, Switzerland). Phospho*enol*pyruvate (PEP) and nicotinamide adenine dinucleotide (reduced) salt (NADH) were purchased from Chem-Impex International Inc. (Wood Dale, IL, USA). All other reagents were of the highest quality commercially available.

### 2.2. Sarcoplasmic Reticulum Isolation

Muscle tissue was obtained from wild-type *Oryctolagus cuniculus* from a local animal handling unit (INE/CITES/DGVS-ZOO-E0055-SLP-98). Sarcoplasmic reticulum vesicles (SRVs) were prepared as described by Champeil et al. (1978) [38], with slight modifications [26]. Briefly, fast-twitch muscles were macerated in a Oster Pro blender (Sunbeam Products Inc., Miami, FL, USA) in three volumes of 100 mM KCl, with 1 min on and 1 min in ice-cold rest repeated three times. Tissue debris was removed using centrifugation (1532× *g*) at 4 °C for 5 min; a second centrifugation (4256× *g*) was performed if necessary. The supernatant was collected and homogenized using a tissue grinder and WiseStir HS-30E (Daihan Scientific Co., Seoul, Republic of Korea), then centrifuged (10,048× *g*) at 4 °C for 15 min. The resulting supernatant containing sarcoplasmic reticulum was centrifuged (33,110× *g*) at 4 °C for 120 min. The pellets were suspended in 0.5 M sucrose and centrifuged (12,111× *g*) at 4 °C for 15 min. The supernatant was diluted to 0.6 M KCl and 0.15 M sucrose and centrifuged (34,310× *g*) at 4 °C for 165 min. The SRVs sheets were suspended in 0.3 M sucrose, 0.1 M KCl, and 5 mM Tris-HCl, pH 7.0. Protein concentration was determined using the Lowry assay [39,40], using human serum albumin as the protein standard. The SRVs (30–32 mg/mL) were aliquoted in 1 mL volumes and stored at −72 °C until use.

### 2.3. SERCA Purification

Sarcoplasmic reticulum Ca^2+^-ATPase (SERCA) was purified using the method described by Sampedro et al. (2007) [36], with slight modifications; the procedure shows high efficiency in purifying yeast plasma membrane H^+^-ATPase [36,41]. Briefly, SRVs were diluted by up to 2 mg/mL of protein in ice-cold 75 mM Tris-HCl, pH 7.2, 0.6 M KCl, 6 mM EDTA, 1 mM EGTA and 0.1% (*w*/*v*) deoxycholate. The mixture was incubated on ice for 10 min with gentle agitation and then centrifuged (103,000× *g*) at 4 °C for 60 min. Pellets were suspended in 25 mM Tris-HCl, pH 7.5, 0.3 M KCl, 45% glycerol (*v*/*v*) and 2 mM EDTA and homogenized. The protein concentration was determined and 5 mg/mL azolectin and 0.85 (*w*/*w*) Zwittergent 3–14 were added. The mixture was homogenized and then centrifuged (103,000× *g*) at 4 °C for 60 min. The supernatant was collected and diluted 1:2 with 2 mM EGTA (pH 7.2). The suspension was then gently poured onto a discontinuous trehalose concentration gradient (45, 40, 35 and 30% *w*/*v*) in 10 mM Tris-HCl, pH 7.0, 1 mM EDTA, 0.1% deoxycholate and 1 mg/mL azolectin. The samples were centrifuged (103,000× *g*) at 4 °C for 14 h. After centrifugation, a transparent slightly yellowish pellet was obtained. The pellet was gently resuspended in a small volume (<1.5 mL) of 25 mM Tris-HCl, pH 7.5, 0.3 M KCl, 45% glycerol and 2 mM EDTA. The protein concentration was determined as described above and then adjusted to 2 mg/mL. Aliquots (~50 µL) were taken and stored at −72 °C until its use.

### 2.4. Enzyme Kinetics

ATPase activity was measured at 37 °C using an enzyme-coupled assay, as previously described [41,42]. The reaction mixture (1 mL) consisted of 50 mM MOPS (pH 7.0), 1 mM EGTA, 80 mM KCl, 5 mM MgCl_2_, 3 mM CaCl_2_, 5 mM phosphoenolpyruvate, 250 μM NADH, and ATP concentration as indicated (0.001 to 0.25 mM). The reaction solution was homogenized carefully by vortexing and then incubated at 37 °C for 10 min. Then, 0.9 U L-lactate dehydrogenase (LDH) and 1.5 U pyruvate kinase (PK) were added and the ATPase reaction was initiated by the addition of 10 μg of the purified SERCA. The NADH formed was monitored by the change in absorbance intensity at a wavelength (λ) of 340 nm using an 8453U*V*/*V*IS spectrophotometer (Agilent Technologies, Waldbronn. DE) equipped with a thermostatted cell holder. The rate of ATP hydrolysis was calculated from the slope of the linear portion of each curve using the molar extinction coefficient (ε) of NADH (λ = 6220 M^−1^·cm^−1^). Velocity data were fitted to the Michaelis–Menten Equation (1) (Equation (1)) using non-linear regression with the Origin 6.0 software:

(1) $$\nu =\frac{V_{max}\cdot \left[S\right]}{K_{m}+\left[S\right]}$$

where *v* is the velocity, *V*_max_ is the maximum velocity, [*S*] is the ATP concentration and *K*_m_ is the Michaelis–Menten constant.

### 2.5. SERCA Labeling with Fluorescein Isothiocyanate (FITC)

Covalent labeling of SERCA with FITC was performed as previously described with slight modifications [43,44,45]. Briefly, purified SERCA (20 μg) was suspended in 50 μL (final volume) labeling buffer (100 mM KCl, 5 mM MgCl_2_, and 30 mM Tris-HCl, pH 8.9) containing 1 mM FITC. After mixing using vortexing, the samples were incubated for different times (15, 10, 7, 5, and 2 min) in the dark at room temperature, and labeling reaction was stopped with 1 volume of ice-cold stopping buffer (480 mM sucrose and 48 mM MOPS, pH 7.0), containing ATP (5 mM); the mixture was incubated on ice for 5 min in the dark. FITC-labeled SERCA was then subjected to SDS-PAGE. The clear gels were exposed to UV light (λ = 302 nm) and photodocumented using a benchtop UV transilluminator (Cole-Parmer, Vernor Hills, IL, USA) and a Coolpix B500 camera (Nikon Corp., Tokyo, Japan). After photographic documentation, the gel was stained with Coomassie blue.

### 2.6. Circular Dichroism (CD) Spectrum of SERCA

Purified SERCA (3 μM) was suspended in 400 μL of 10 mM phosphate buffer (pH 7.0) at 25 °C. Aliquots of ATP were added stepwise as indicated (5–100 μM final concentration) in the absence and presence of Ca^2+^ (200 μM). Far-UV spectra (λ range of 190–260 nm) were recorded at 50 nm/min in a Jasco J1500 spectropolarimeter (Jasco Inc., Tokyo, Japan) using a 0.1 cm path length cell at 25 °C. The internal resolution of the data was 1 nm and the bandwidth was 1 nm.

## 3. Results

### 3.1. SERCA Purification

SERCA is the most abundant protein (~90%) embedded in the SR membrane [46]. Therefore, the purification of SERCA seems straightforward and significant material would be expected to be obtained. As a result, numerous isolation methods for SERCA have been reported, with variable protein yields, but showing ATP hydrolysis dependent/coupled to Ca^2+^ ion transport [7,12,38,44,47,48,49,50,51,52,53]. However, the instability of SERCA during handling (purification, storage, and experimental) is a problem [12,44,52,53,54]; e.g., the intrinsic fluorescence intensity of SERCA gradually decreases during incubation time [29,30,55,56]. Preservation of the native functional state is important for structural and functional studies of this ATPase (and any other protein) [30,31,32,57].

**Figure 1:**
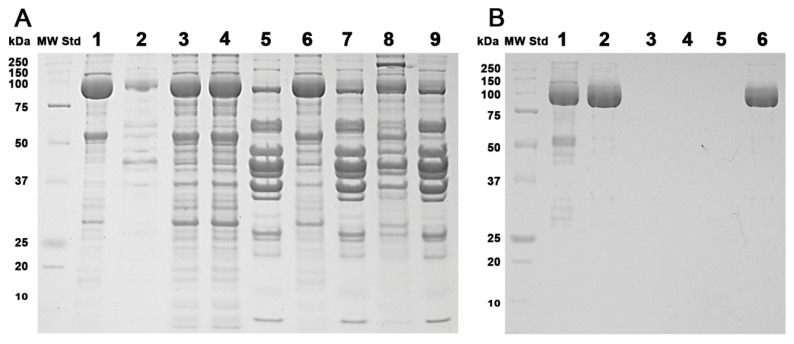
Purification of SERCA from rabbit fast-twitch muscle. (**A**) SDS-PAGE of SRVs purification at different stages of differential centrifugation; protein was visualized using Coomassie blue staining. Lanes: 1, purified SRVs (pellet 5); 2, supernatant (centrifugation 5); 3 and 4, supernatant and pellet (centrifugation 4); 5 and 6, supernatant and pellet (centrifugation 3); 7 and 8, supernatant and pellet (centrifugation 2); 9, supernatant (centrifugation 1). (**B**) Isolation of SERCA using ultracentrifugation on a discontinuous trehalose gradient. Purified SRVs were treated as described in Methods. Purified SERCA was treated as in (**A**) for protein visualization. Lanes: 1, purified SRVs; 2, purified SERCA (pellet); 3, 4, and 5: samples at 45, 40, and 35% trehalose (*w*/*v*); 6, SERCA aggregates after ultracentrifugation in the absence of trehalose. All lanes were loaded with 20 µg of protein, except in B where lanes 3, 4, and 5 were loaded with ~6.2 µg each.

In this work, the purification of rabbit fast-twitch-muscle SERCA was carried out using the purification methodology used for the isolation of yeast plasma membrane H^+^-ATPase, as it allows the successful isolation of the functional hexameric state [31]. Sarcoplasmic reticulum vesicles (SRVs) were obtained, and the kinetic parameters (*K*_m_ and *V*_max_) for ATPase activity of the isolated SRVs were similar to those reported in the literature (*V*_max_ = 7.0 µmoles ATP/min·mg protein and *K*_m_ = 15.1 µM ATP) [26]. The purification of SERCA from SRVs was then performed as described in the Methods [36]; SRVs (SERCA ~110 kDa) at different steps of isolation were monitored using SDS-PAGE (Figure 1A). SRVs were solubilized with detergent and importantly, after centrifugation (103,000× *g*) on a trehalose concentration gradient, a clear pellet was observed at the bottom of the centrifuge tubes containing SERCA in high purity (Figure 1B). Samples at different tube heights were also taken and analyzed. Notably, SERCA was only observed in the pellet (Figure 1B). The purity of SERCA was ≈95%, as determined using densitometry [31]. Importantly, when SERCA was ultracentrifuged in the absence of trehalose, it denatured, forming white insoluble aggregates and irreversibly losing ATPase activity (Figure 1B, last lane). The isolation protocol was tested at least three times with similar results for protein purity. Suspended SERCA was stored at −71 °C until use.

### 3.2. ATPase Kinetics

**Figure 2:**
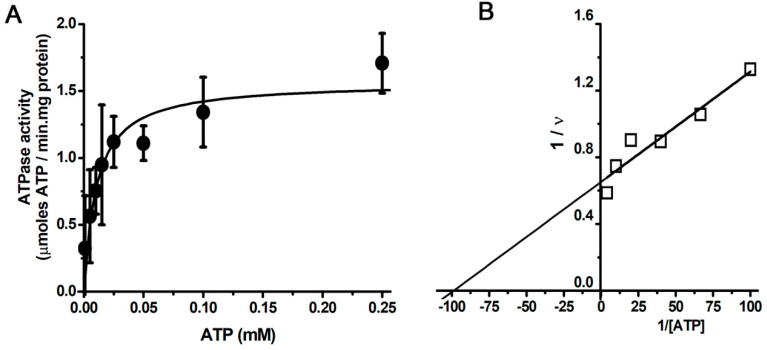
Enzyme kinetics of isolated SERCA. The assay for ATPase activity was performed at 37 °C using an enzyme−coupled assay (SERCA−PK−LDH) in buffer 50 mM MOPS, pH 7. (**A**) Dependence of SERCA specific activity (●) on ATP concentration. Data points represent the mean of three independent experiments plus or minus standard deviation. The rate of ATP hydrolysis showed a hyperbolic dependence on ATPase kinetics, so the data were fitted to the Michaelis–Menten equation (Equation (1)) using non−linear regression (*V*_max_ = 1.68 ± 0.09 µmoles ATP/min·mg protein, and *K*_m_ = 12.16 ± 2.25 µM ATP). (**B**) Lineweaver–Burk plot of the data in (**A**) (≤).

The rate of ATP hydrolysis (37 °C) of the purified SERCA showed a hyperbolic pattern of dependence on ATP concentration (Figure 2A). The specific activity was in agreement with that reported by Shivanna and Rowe (1997) [58]. The rate data for ATP hydrolysis were fitted to the Michaelis–Menten equation (Equation (1)) using non-linear regression, and the kinetic parameters were calculated (Figure 2): *V*_max_ = 1.68 ± 0.09 µmoles ATP/min·mg protein, and *K*_m_ = 12.16 ± 2.25 µM ATP. In the literature, the published *V*_max_ of SERCA purified using different methodologies ranges from 0.8 to 4.5 µmol ATP/min · mg protein [56,59,60,61,62,63,64,65]. Notably, the *K*_m_ value was similar to that determined for isolated SRVs (*K*_m_ = 15.1 ± 2.1 µM ATP), and to that reported in the literature [30,66,67,68]. Certainly, the native SR membrane environment is ideal for maximum ATPase activity [58].

### 3.3. FITC Labeling of SERCA

**Figure 3:**
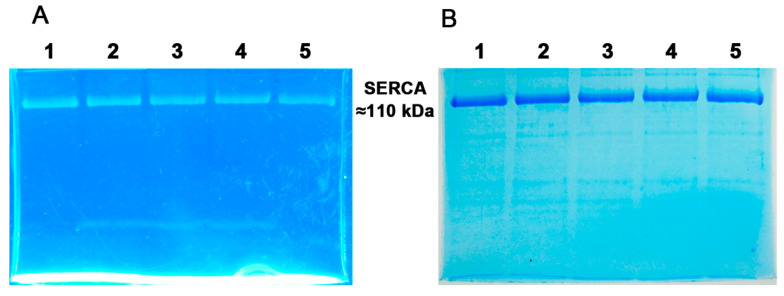
(**A**) FITC labeling of SERCA. Purified SERCA was incubated in the presence of FITC for different times and then subjected to SDS-PAGE; lanes: 1, 15 min; 2, 10 min; 3, 7 min; 4, 5 min; 5, 2 min. The clear gel was exposed to UV light (λ = 302 nm) and photo-documented. Free FITC is at the bottom of the gel. (**B**) Coomassie blue staining of the gel in (**A**). The lanes were loaded with 20 µg of SERCA.

In SERCA, FITC-labeling indicates the intactness and interacting functionality of the ATP-binding site [26,37,43,44]. FITC interacts with the nucleotide binding site of the enzyme [37], resulting in the formation of a covalent bond between FITC and a lysine (Lys) residue [37,45,69,70]. SERCA was subjected to FITC labeling at different incubation times as described in the Methods, followed by SDS-PAGE and photo-documentation using UV irradiation (Figure 3A). The gel in Figure 3A shows a single bright green fluorescent band corresponding to the molecular weight of SERCA (MW ~110 kDa) (Figure 3A). The gel was then stained with Coomassie blue (Figure 3B). The resulting blue bands (~110 kDa) were superimposed on those showing green fluorescence under UV irradiation (Figure 3A). FITC labeling was also performed in the presence of 3 mM Ca^2+^, but no change in FITC labeling was observed. FITC labeling occurred relatively rapidly, as no significant differences were observed at the incubation times tested (Figure 3).

### 3.4. Secondary Structure of SERCA

**Figure 4:**
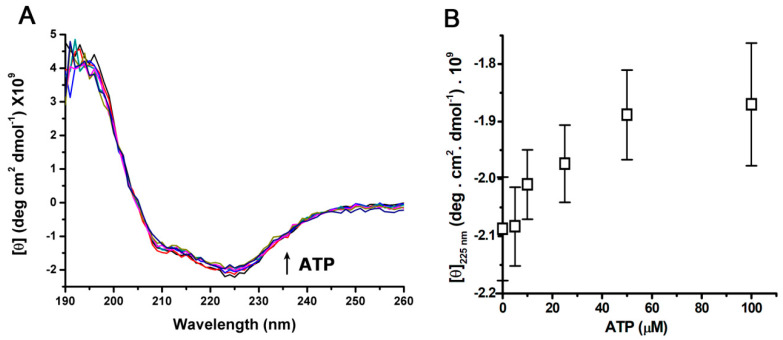
Effect of the presence of ATP on the circular dichroism (CD) spectrum of isolated SERCA. (**A**) CD spectra of SERCA at 25 °C. ATP was added stepwise to a SERCA suspension, and then mixed, and a CD spectrum was obtained (three accumulations); the assay was repeated three times, and a representative experiment is shown. (**B**) Effect of ATP on CD intensity at λ of 225 nm. Data show mean and standard deviation from experiments in (**A**).

The secondary structure of SERCA was analyzed using circular dichroism (CD) spectroscopy. SERCA (3 µM) was suspended in 10 mM phosphate buffer (pH 7.0), and the circular dichroism (CD) spectrum was recorded at 25 °C (Figure 4A). ATP was then added stepwise (0–100 µM) and CD spectra were recorded after each addition. The results showed a slight change in molar ellipticity as a function of ATP concentration at a wavelength (λ) of 225 nm (Figure 4A), but not at λ of 212 nm. A hyperbolic pattern was observed at ATP saturation (Figure 4B), which was similar to that observed in a recombinant engineered Ca^2+^-ATPase N-domain [37]. Notably, the secondary structural changes in α-helices mediated by the presence of ATP occurred in the μM region for the ATP affinity determined by enzyme kinetics (Figure 2). Therefore, the change in CD intensity at λ of 225 nm appears to correspond to changes in secondary structure (α-helices) induced by ATP binding [71]. The experiment was repeated in the presence of 200 µM Ca^2+^, but no significant differences in the CD spectra (200–260 nm) were observed, in agreement with that reported by Csermely et al. (1987), and by Shivanna and Rowe (1997) [58,71].

## 4. Discussion

Most integral membrane proteins/enzymes are difficult to isolate in their native functional state due to their hydrophobic nature [72]. The detergent solubilization of biological membranes usually leads to the disruption of embedded macromolecular complexes, dissociation of protein/enzyme oligomers, protein unfolding, and consequently the loss of function/activity [58]. Any effective method of isolating membrane proteins must minimize this disruption of the delicate structural arrangement.

The use or inclusion of cosmotropic agents in isolation buffers can help to improve the retention of the native structure and, thus, the function of proteins [30,73,74]. In this sense, the disaccharide trehalose is a well-known protein stabilizer synthesized by yeast, plants, and other organisms under harsh conditions such as heat shock temperatures, desiccation, and others [75,76,77,78,79,80,81,82,83,84,85,86,87]. Various studies have shown that trehalose is superior in protein stabilization when compared to other saccharides and polyols [32,33,88,89]. The molecular mechanism by which trehalose stabilizes and protects the three-dimensional structure of proteins is essentially well understood [33,34,88,90,91,92]. With regard to P-ATPases, the disaccharide trehalose has been shown to be able to stabilize H^+^-ATPase under various incubation/storage conditions (mainly dehydration and heat) [32,33], but also during the H^+^-ATPase purification process [31], i.e., the presence of trehalose prevents the loss of the native structure during H^+^-ATPase isolation, thus allowing macromolecular complexes formed by the enzyme hexamer to be isolated [31]. The oligomeric state of the H^+^-ATPase is known to be highly labile. In plants, the H^+^-ATPase hexamer is stabilized by the fungal toxin fusicoccin via 14-3-3 protein binding [93]. In yeast, trehalose appears to prevent the dissociation of the H^+^-ATPase hexamer, thus avoiding the loss of its quaternary structure and ATPase activity [31,33]. Notably, the 3D structure of the Ca^2+^-ATPase in its dimeric state is currently lacking; similarly, the oligomeric structure of other P-type ATPases remains to be determined.

In this work, SERCA was purified in both a relatively high yield and purity by incorporating trehalose as a protein stabilizer. Trehalose is useful in preserving both structure and function, as evidenced by results obtained from FITC labeling, secondary structure analysis (CD spectrum), and ATPase activity. This protocol offers advantages when compared to published molecular biology protocols; e.g., it does not require additional reagents, material, and processing enzymes (for gene cloning and protein expression and purification) or equipment, and mainly, the protein purified is the native enzyme. In regard to its disadvantages, the presence of other native residual proteins may be a problem. Thus, the SERCA isolation method described here will allow future structural/functional/regulatory studies, for example (a) to understand of functional divergence of isozymes [94] (i.e., the functional role of amino acid sequence variation) and the regulation of activity [15]; (b) the structural–mechanistic defect in mutation-mediated diseases [95,96,97,98,99,100]; (c) to test new compounds with inhibitory properties in a given Ca^2+^-ATPase pump (e.g., cardiac) [101], i.e., the identification of the structural target, and mechanism of inhibition of natural or newly designed inhibitors [101]; (d) the testing of molecular docking and molecular dynamics simulation results; (e) the clarification of the ion transport mechanism [48]; and (f) the role of specific structural dynamics in its function. The high isolation efficiency (~95%) of SERCA and PMA1 are examples of improving the effectiveness of the purification methodology developed in our laboratory [31,36]. In addition, it is expected that the protocol will be applied to efforts to purify other P-ATPases and unstable proteins (cytosolic and integral membrane).

## Data Availability

The data presented in this study are available in this article.
